# Evaluation of Early Concrete Damage Caused by Chloride-Induced Steel Corrosion Using a Deep Learning Approach Based on RNN for Ultrasonic Pulse Waves

**DOI:** 10.3390/ma16093502

**Published:** 2023-05-01

**Authors:** Julfikhsan Ahmad Mukhti, Kevin Paolo V. Robles, Keon-Ho Lee, Seong-Hoon Kee

**Affiliations:** 1 Department of ICT Integrated Ocean Smart Cities Engineering, Dong-A University, Busan 49304, Republic of Korea; julfikhsanam@donga.ac.kr (J.A.M.); kpvrobles@donga.ac.kr (K.P.V.R.); 2 Department of Architectural Engineering, Dong-A University, Busan 49304, Republic of Korea; 3 National Core Research Center for Disaster-Free and Safe Ocean Cities Construction, Dong-A University, Busan 49304, Republic of Korea

**Keywords:** concrete deterioration, non-destructive evaluation, ultrasonic testing, deep learning, recurrent neural network

## Abstract

The objective of this study is to explore the feasibility of using ultrasonic pulse wave measurements as an early detection method for corrosion-induced concrete damages. A series of experiments are conducted using concrete cube specimens, at a size of 200 mm, with a reinforcing steel bar (rebar) embedded in the center. The main variables include the water-to-cement ratio of the concrete (0.4, 0.5, and 0.6), the diameter of the rebar (10 mm, 13 mm, 19 mm, and 22 mm), and the corrosion level (ranging from 0% to 20% depending on rebar diameter). The impressed current technique is used to accelerate corrosion of rebars in concrete immersed in a 3% NaCl solution. Ultrasonic pulse waves are collected from the concrete specimens using a pair of 50 kHz P-wave transducers in the through-transmission configuration before and after the accelerated corrosion test. Deep learning techniques, specifically three recurrent neural network (RNN) models (long short-term memory, gated recurrent unit, and bidirectional long short-term memory), are utilized to develop a classification model for early detection of concrete damage due to rebar corrosion. The performance of the RNN models is compared to conventional ultrasonic testing parameters, namely ultrasonic pulse velocity and signal consistency. The results demonstrate that the RNN method outperforms the other two methods. Among the RNN methods, the bidirectional long short-term memory RNN model had the best performance, achieving an accuracy of 74% and a Cohen’s kappa coefficient of 0.48. This study establishes the potentiality of utilizing deep learning of ultrasonic pulse waves with RNN models for early detection of concrete damage associated with steel corrosion.

## 1. Introduction

**Figure 1:**
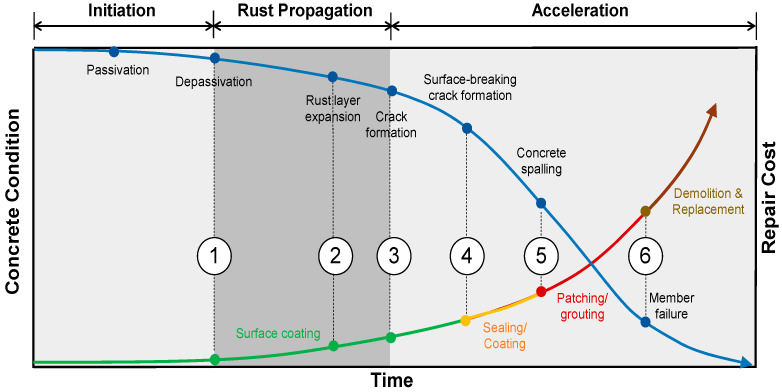
Conceptual illustration of concrete deterioration caused by corrosion of steel in concrete and cost for rehabilitation of the deteriorated concrete with various severities [14]. Deterioration stages: ① rebar de-passivation; ② rust layer expansion; ③ crack formation; ④ surface-breaking crack formation; ⑤ concrete spalling; and ⑥ member failure.

Chloride-induced corrosion of steel is one of the most significant sources of deterioration in reinforced concrete structures [1,2,3]. It has been known that the chloride-induced deterioration mechanism occurs in three phases: corrosion initiation, rust propagation, and corrosion acceleration, as illustrated in Figure 1 [4,5,6]. The corrosion initiation process starts when the thin passive layer (Fe_2_O_3_) on the surface of the reinforcing steel bar (rebar) becomes unstable and depleted due to the migration of chloride ions [7,8]. The rust then propagates and begins to form on the rebar surface. This oxidation process of the metallic iron causes the rebar volume to increase up to about sixfold on the oxidized section compared to its uncorroded state [9]. This increasing volume induces internal tensile stress on the concrete, which could result in enhanced porosity and microcracks in the steel and concrete interfaces [10]. As the tensile stress increases, the microcracks start to spread and open more paths for chloride ions to penetrate the concrete [11], increasing the permeability of the concrete. This also accelerates the penetration rate of harmful substances and moisture within the concrete and the corrosion process. Without sufficient maintenance activities, this could lead to major damages such as surface-breaking cracks, spalling, and delamination defects, which affect the integrity of the structure [12]. Moreover, beyond the point of corrosion acceleration, the cost of rehabilitation of reinforced concrete elements would exponentially increase after the formation of visible concrete damages such as surface-breaking cracks and spalling [13] (see Figure 1). Therefore, it is important to evaluate the condition of reinforced concrete exposed to harsh environmental conditions on corrosion of steel in concrete and, if necessary, to perform appropriate preventive maintenance actions.

It is known that microscopic changes in corrosion-induced concrete damages can be effectively evaluated by several laboratory testing techniques such as scanning electron microscopy (SEM), nuclear magnetic resonance (NMR), and thermo-gravimetric/derivative thermo-gravimetric (TG/DTG) [15]. However, these methods require an invasive procedure to obtain testing samples for laboratory inspection and inevitably induce some surface damages in concrete. Furthermore, these methods could include additional processes (e.g., surface preparation, sampling, and repairing) when applied to actual structures, which makes the methods labor-intensive and high-priced. Therefore, invasive testing methods are reluctantly used for condition assessment of actual structures.

There are various non-destructive evaluation (NDE) techniques that are effective for in situ evaluation of chloride-induced steel corrosion in concrete structures. The corrosion of steel in concrete can be explained by an electrochemical process in which both flows of electrical currents and chemical reactions occur. NDE methods based on electrochemical principles have been widely used to evaluate the initiation and activity of steel corrosion in concrete. For example, the half-cell potential (HCP) measurement is used to investigate the electrical activity of steel corrosion in concrete induced by chloride ions [16]. The probability of corrosion activity of steel in concrete is determined based on HCP (or corrosion potential, *E_corr_*) readings in accordance with ASTM C876-15. Furthermore, the rate of steel corrosion (or corrosion current density, *i_corr_*) can be measured by the polarization resistance of steel in concrete, *R_p_*, which is directly proportional to *i_corr_*. There are several NDT methods for measuring the polarization resistance of steel in concrete: linear polarization resistance method [17], Tafel extrapolation method [18], electrochemical impedance spectroscopy [19], etc. The electrical resistivity (ER) measurement has also been widely studied for evaluating the corrosive environment (e.g., water saturation and chloride penetration) of concrete and the corrosion rate of steel in concrete [20]. However, it has been challenging to evaluate the damage of concrete associated with steel corrosion by electrochemical measurement parameters such as HCP (or *E_corr_*), *R_p_*, *i_corr_*, and/or ER. Those parameters could be highly affected by various environmental factors (e.g., water saturation, humidity, and temperature) which are not really correlated with concrete deterioration (e.g., enhanced porosity, microcracks, and surface-breaking cracks).

**Figure 2:**
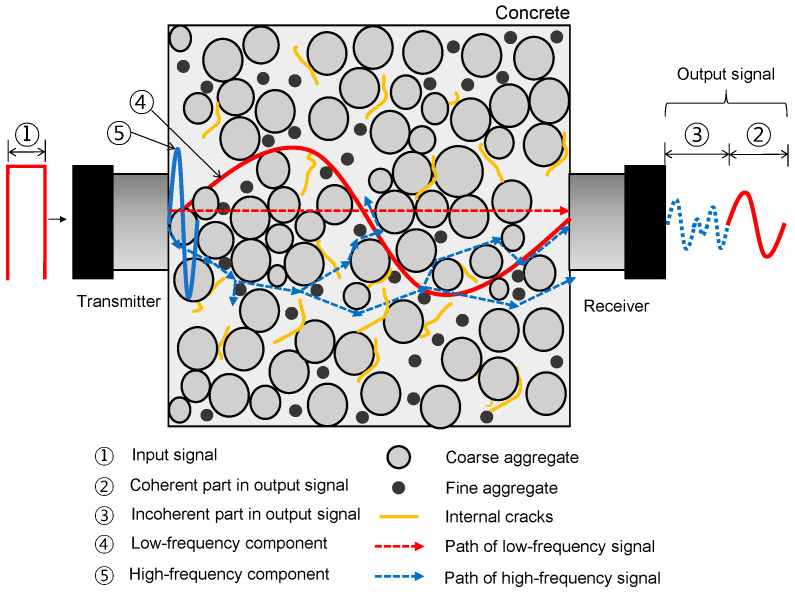
Illustration of the ultrasonic pulse wave propagation through concrete, a heterogenous and anisotropic material.

On the other hand, it has been demonstrated by numerous researchers that ultrasonic pulse wave measurements are effective for evaluating the various types of concrete deteriorations such as honeycombing [21], delamination defects [22], surface-breaking cracks [23,24], microcracks [25], and bottom-up cracks [26]. Figure 2 illustrates the ultrasonic pulse wave propagation through concrete, a heterogenous and anisotropic material. A transmitting transducer, placed on one side of the concrete, generates ultrasonic pulse waves travelling through the concrete, which are measured by a receiving transducer on the opposite side of the concrete. Concrete acts as a low-pass filter for ultrasonic pulse waves. Some low-frequency components directly propagate to the receiver, while some high-frequency components are suppressed and/or delayed by reflection and/or diffusion due to the heterogeneous and anisotropic features of concrete. Theoretically, the earlier part (also called the coherent part) of the ultrasonic signals is informative of the global properties of concrete, while the later part (also called the incoherent part) of the signals is a result of the superposition of the diffused waves from the presence of aggregates and various defects in concrete [27]. Several previous researchers have used the coherent part of the ultrasonic pulse waves to evaluate the properties of concrete [20,22,27]. For example, it has been demonstrated that ultrasonic pulse velocity (UPV) of concrete is a good indicator of the overall quality of concrete with various deterioration levels. The presence of defects in concrete would delay the first arrival time (time of flight) of the ultrasonic pulse waves through concrete [27]. UPV has been demonstrated to be sensitive to major faults or open fractures, which serve as an effective barrier to ultrasonic wave transmission. However, it is known that UPV is unlikely to be affected by such minor faults as enhanced porosity and/or micro-, ill-defined, and closed cracks in concrete, which are generated by steel corrosion in concrete.

Some researchers found that the signal interpretation based on the change in the incoherent part is more effective for evaluating the early concrete damages using nonlinear ultrasonic parameters such as coda wave interferometry (CWI), sideband peak count index (SPC-I), and energy redistribution index [25]. Schurr et al. [28] used CWI to detect small-scale concrete damages caused by external loadings. In their research, the change in the phase shift of the incoherent part of ultrasonic signals is far clearer than that of the coherent part. Research by Castellano et al. [29] observed that more cracks and voids in concrete, caused by cyclic loadings, increased the SPC-I, the number of peaks above a threshold in the spectral amplitude of ultrasonic signals. It was demonstrated that SPC-I is sensitive to minor concrete damages caused by the early load steps, while UPV values remained stable. Furthermore, Arumaikani et al. [24] noticed that the SPC-I is effective in evaluating internal concrete damages caused by corrosion of steel in concrete that could not be observed on the surface of the concrete. However, it has been argued that the performance of the evaluation model based on such nonlinear ultrasonic parameters can be strongly dependent on engineering judgments. As will be discussed in this study, the sensitivity of the nonlinear parameters could be affected by the choice of nonlinear parameters and input signals used for the calculation of the parameters.

Another, more systematic solution for exploiting ultrasonic pulse waves to detect early concrete damage is the use of deep learning. Since ultrasonic pulse signals are time series, the data sequence is an important feature, and the recurrent neural network (RNN) would be a good method for deep learning of ultrasonic pulse waves. The RNN method has been successfully used in several fields, most notably in medicine. Singh et al. [30] utilized the RNN models for the classification of electrocardiogram (ECG) data for detecting arrhythmia with an accuracy of up to 88.1%. Kim et al. [31] utilized both RNN and convolutional neural network (CNN) methods for the classification of ECG data. In their study, the RNN models perform slightly better than CNN. Additionally, the RNN method has also been successfully applied for more complex signals such as human speech. Rejaibi et al. [32] applied the RNN method for differentiating human voices in depressed and non-depressed states with more than 70% accuracy. However, the application of RNN to monitor concrete conditions based on ultrasonic pulse data is currently very limited.

The main objective of this research is to investigate the feasibility of ultrasonic pulse wave measurements as an early detection method for corrosion-induced concrete damages by using a deep learning classification model based on RNNs. For these purposes, this study aims to perform three main tasks as follows: (1) evaluating the change of parameters in the ultrasonic pulse signal from each method to corrosion levels, which will be undertaken by performing accelerated corrosion to reinforced concrete specimens, (2) developing classification models through deep learning of ultrasonic pulse waves based on three RNN algorithms (long short-term memory, gated recurrent unit, and bidirectional long short-term memory), and (3) developing classification models based on conventional ultrasonic testing parameters (ultrasonic pulse wave velocity and signal coherence). This study will demonstrate the potential of deep learning classification models based on RNN of ultrasonic pulse waves for early detection of concrete damages, which is superior to the classification models based on the conventional ultrasonic testing parameters.

## 2. Materials and Methods

### 2.1. Fabrication of Test Specimens

**Figure 3:**
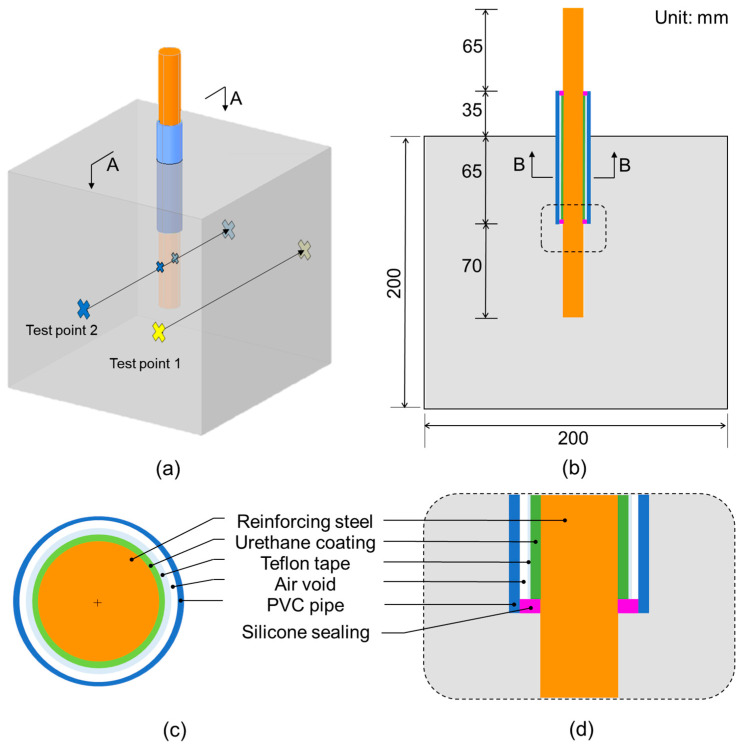
Illustration of a reinforced concrete cube specimen used in this study: (**a**) isometric view, (**b**) section AA in (**a**), (**c**) section BB in (**b**), and (**d**) detail in a dashed box in (**b**).

Figure 3 illustrates a reinforced concrete cube specimen with a size of 200 × 200 × 200 mm^3^ used in this study. A 235 mm long reinforcing steel bar (rebar) was embedded in the middle of the concrete cube specimen (see Figure 3b). A middle part of the rebar was waterproofed by three layers of coating (see Figure 3c): first, two thin layers of urethane were applied on the surface of the rebar; second, Teflon tape was rounded on the hardened urethane layer; third, a 100 mm long PVC pipe was placed on the Teflon coating in the middle of the rebar. Consequently, only 70 mm of the rebar was directly exposed for accelerated corrosion. The concrete cubes were shaped using 20 mm thick wooden forms. Concrete was cast as the rebar was horizontally situated through a punch-hole on one side of the forms. Concrete specimens were cured in the air for 24 h after casting concrete. After demolding, the concrete specimens were moved to and stored in a constant temperature and humidity room in the laboratory (temperature of 20 ± 3 °C and relative humidity of 50 ± 5%). The concrete cube specimens were divided into four groups based on nominal diameters of rebars in concrete: 10 mm, 13 mm, 19 mm, and 22 mm, which are referred to as D10, D13, D19, and D22, respectively. Each group has three sub-groups with three different design compressive strengths of concrete: 18 MPa, 24 MPa, and 40 MPa. Table 1 shows the mixture proportions of the concrete used for the fabrication of the concrete cubes. Rebars in the concrete specimens were subjected to various corrosion levels. The target corrosion levels in this study were 0%, 3%, 6%, and 12% for D10 and D13 specimens and 0%, 5%, 10%, and 20% for D19 and D22 specimens. In addition, there were three copies of concrete cube specimens for each combination of test variables. Consequently, this study included a total of 108 reinforced concrete cube specimens.

### 2.2. Accelerated Corrosion Tests

**Figure 4:**
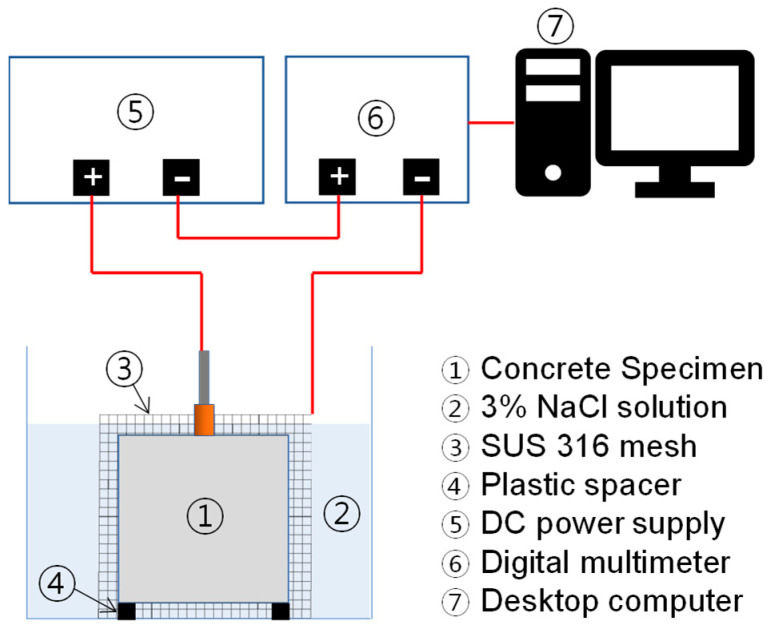
Test setup for the accelerated corrosion test by the impressed current technique.

The rebars in concrete cube specimens were subjected to an accelerated corrosion process to simulate the chloride-induced steel corrosion using the impressed current technique. The target theoretical corrosion levels in this study were calculated based on the following Faraday’s Law [33]:

(1) $$M_{th}=\frac{WI_{app}T}{F}$$

where $M_{th}$ is the theoretical mass density of steel rust (kg/cm^2^), *W* is the equivalent weight of steel as a ratio of the atomic iron weight to the iron valency (27,925 g), *I_app_* is the current density applied to the specimen (A/cm^2^), *T* is the duration of current flows (or corrosion process) (s), and *F* is Faraday’s constant (~96,487 As). Before the accelerated corrosion process, each specimen was immersed in a 3% NaCl solution until fully saturated condition. Then, the specimen was electrified on a setup shown the Figure 4. The positive pole of the power supply (Sorensen XPF 60-20D) was attached to the rebar, which effectively made the rebar an anode. The negative pole of the power supply was connected to the stainless-steel mesh (i.e., SUS 316) around the specimen, which served as a cathode. The corrosion of the rebars in the concrete specimens started as soon as the current was sent by the power supply. A digital multimeter (Keysight 34461A) was located between the power supply and the stainless-steel mesh to monitor the current flowing through the concrete specimens. The current data measured by the digital multimeter were stored in a desktop computer through a LabVIEW-based monitoring program and the theoretical corrosion level of rebar was automatically calculated in real time in the program. The accelerated corrosion test for each specimen continued until the target theoretical corrosion levels were reached.

### 2.3. Steel Mass Loss Ratio

In this study, the steel mass loss ratio of corroded rebars, *θ* (i.e., corrosion level in this study), was defined as the ratio (in percentage) of the mass loss of the corroded rebar, $m_{s,loss}\left(\theta \right)$, normalized by the mass of the solid rebar in the working area, $m_{s,WA}\left(0\right)$, as follows:

(2) $$\theta =\frac{m_{s,loss}\left(\theta \right)}{m_{s,WA}\left(0\right)}\times 100\;\left[\%\right]$$

**Figure 5:**
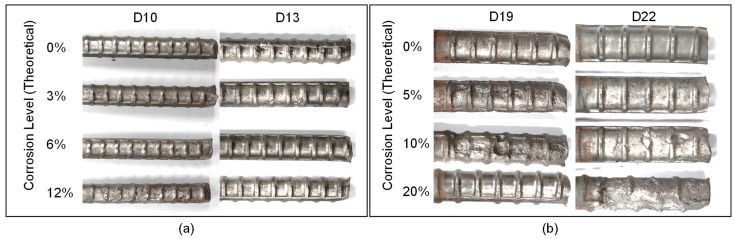
Rebar condition after corrosion for (**a**) D10 and D13 specimens, and (**b**) D19 and D22 specimens, respectively.

The actual amount of steel mass loss was evaluated in accordance with ASTM G1-03 [34]. After the accelerated corrosion process finished, the specimens were broken into two parts by using a splitting tensile test setup, and rebars in concrete were separated from the concrete. The corroded rebars were first cleaned by using ultrasonic waves and immersed in NaOH solution to remove the rust layer in steel bars. The sandblasting method was used to remove the remaining rust that was not removed using the mentioned procedure, which resulted in cleaned rebars (see Figure 5).

The actual steel mass loss was determined by Archimedes’s principle of buoyancy. First, the weight of the cleaned rebar was measured in the air, which is denoted as *m*_*s*,*air*_. Second, the cleaned rebar was submerged in the water by 70 mm (the depth of the working area), and the weight of the cleaned rebar was measured, which is denoted as *m*_*s*,*water*_. Then, the mass of corroded rebars corresponding to the working area can be determined as follows:

(3) $$m_{s,WA}\left(\theta \right)=(m_{s,air}\left(\theta \right)-m_{s,water}\left(\theta \right))\rho_{s}/\rho_{w}$$

Here, *θ* is corrosion level of corroded rebars and ρ_s_ and ρ_w_ are mass densities of steel and water, respectively. Then, the steel mass loss of corroded rebars, $m_{s,loss}\left(\theta \right)$, was determined by difference between $m_{s,WA}\left(0\right)$ and $m_{s,WA}\left(\theta \right)$, as follows:

(4) $$m_{s,loss}\left(\theta \right)=m_{s,WA}\left(0\right)-m_{s,WA}\left(\theta \right)\;$$

**Figure 6:**
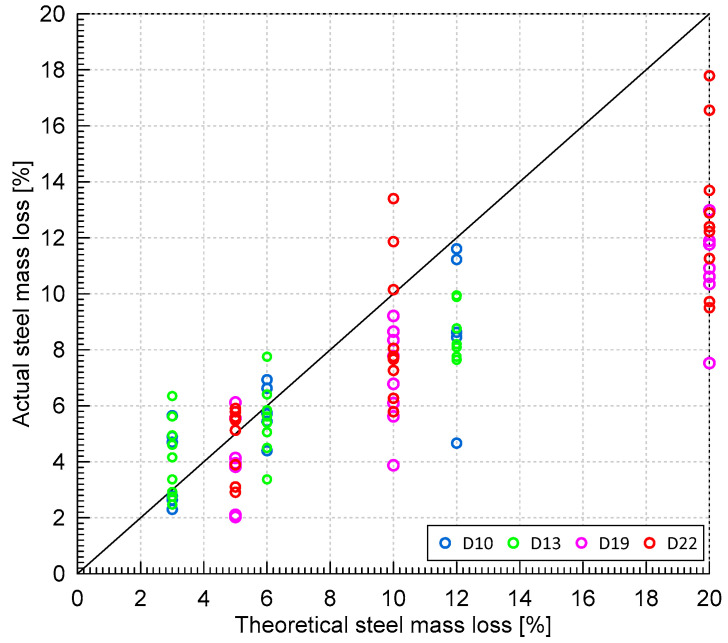
Relationship between the actual (measured) and the theoretical mass loss of reinforcing steel bars with various nominal diameters (D10, D13, D19, and D22).

Figure 6 shows the comparison of theoretical and actual steel loss of the reinforcing steel considering all design mixes and rebar diameters. It can be observed that the theoretical steel loss generally overestimates the actual steel loss. In this study, the actual (or measured) steel loss values were used for correlating the degree of concrete damages and the change in the ultrasonic pulse waves.

### 2.4. Ultrasonic Pulse Wave Measurements

**Figure 7:**
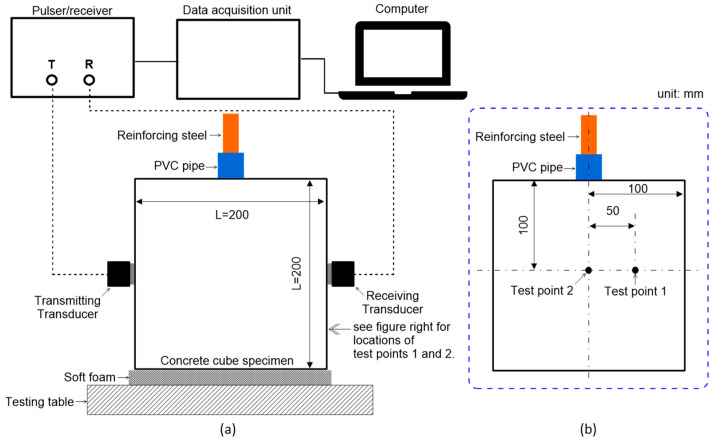
Test setup for ultrasonic pulse wave measurements through the concrete cube specimens: (**a**) source and receiver in the through-transmission configuration, and (**b**) location of test points 1 and 2 on a surface for ultrasonic measurements.

Figure 7 illustrates the test setup of ultrasonic pulse wave measurements transmitted through a reinforced concrete cube specimen. The standard test procedure according to ASTM C 597/C597M-16 [35] was used to measure ultrasonic pulse waves through concrete cube specimens that were subjected to different steel corrosion levels. The setup is composed of a pulser-and-receiver (Olympus 5077PR), a digital oscilloscope (PXIe1073), a pair of P-wave transducers (Olympus X1021), and a desktop computer for data acquisition, display, and storage. The pulser-and-receiver droved the 50 kHz P-wave transducer by a 100 V rectangular pulse with a width of 10 µs. Transducers with a center frequency range of 50–54 kHz are commonly used for NDE based on ultrasonic pulse wave data [36,37], including studies for the detection of internal cracks due to corrosion [24]. The receiving transducer placed on the opposite side of the concrete specimen measured the ultrasonic pulse waves through the concrete. The received signal was digitized with a 10 MHz sampling rate by the digital oscilloscope. Ultrasonic pulse wave measurements were conducted on each specimen before and after the accelerated corrosion test. In this study, ultrasonic tests were performed on the two test points on the surface of ultrasonic measurements (see Figure 3a and Figure 7b). Sensors on test point 1 were attached to the concrete surface directly above the rebar, while sensors on test point 2 were located 50 mm horizontally from the center to avoid the rebar. In this study, five measurements were repeated at each test point.

**Figure 8:**
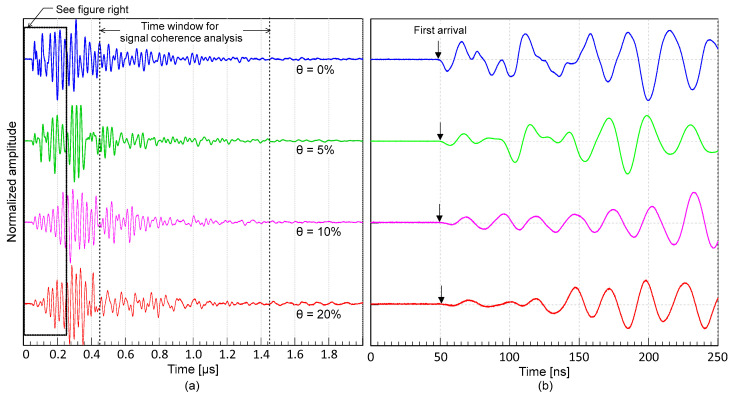
Typical time signals of ultrasonic pulse waves propagating through the D19 concrete cube specimens under the four different target steel corrosion levels: (**a**) ultrasonic waves at different target corrosion levels (θ = 0%, 5%, 10%, and 20%), and (**b**) enlarged ultrasonic signal around the first arrival of the wave shown in (**a**).

Figure 8a presents the typical P-wave signals measured from the concrete cube specimens, and the enlarged signals are shown in Figure 8b. The signals from Figure 8a,b were processed in MATLAB using normalization by Z-scoring. The velocity of an ultrasonic wave can be calculated by dividing the wave path by the travel time, as follows:

(5) $$V_{p}=\frac{L}{t_{a}-t_{d}}$$

where *V_p_* is the wave propagation velocity, *L* is the distance between transducers (200 mm in this study), *t_a_* is the initial wave arrival time, and *t_d_* is the delay time computed during probe calibration. When the two transducers were positioned opposite each other, the time for the first arrival wave was recorded, and the delay time was calculated. It should be noted that P-waves are potentially faster in time signals than any other refracted and reflected waves from the boundary of concrete cube specimens. The arrival of transient stress waves through cylinders was computed using the modified threshold approach based on the observed ultrasonic signals. Using the conventional threshold method used in earlier investigations, an estimated arrival time was initially obtained in this way. After that, a precise arrival time was calculated by fitting a line to the signal data. The intersection of the two P-wave travel times was then used to determine the P-wave travel time. The intersection of the fitting line and the measured zero-signal stage was used to determine the P-wave travel time.

To analyze the nonlinear parameters of ultrasonic wave signals, this study used signal coherence in the form of magnitude square coherence (MSC). The MSC function is calculated by

(6) $$\gamma_{xy}\left(f\right)=\frac{S_{xy}\left(f\right)}{\sqrt{S_{xx}\left(f\right)S_{yy}\left(f\right)}}$$

where $\gamma_{xy}\left(f\right)$ is the coherence, $S_{xy}\left(f\right)$ is the cross-spectral density of *x* and *y*, and $S_{xx}\left(f\right)$ and $\;S_{yy}\left(f\right)$ are the power spectral densities of *x* and *y*, respectively. The resultant value is a number between 0 and 1.0, with a value around 1.0 indicating high signal coherence.

**Figure 9:**
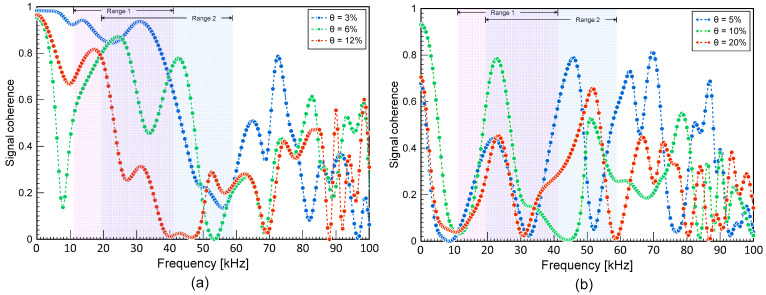
Typical signal coherence curves of ultrasonic pulse waves measured on the MIX1 concrete specimens with various corrosion levels: (**a**) D10 and D13 specimens, and (**b**) D19 and D22 specimens.

As can be seen from the equation, assessing the coherence between concretes with and without damage requires a baseline value. To obtain the coherence values in this study, signals were initially collected on specimens before and after the accelerated corrosion process. A subset of the time-domain signals was then chosen, and each signal was converted into power spectral density using fast Fourier transform (FFT). In this study, a part of the tail end of the ultrasonic wave signals with a length of 0.1 ms was used for signal consistency calculations. The coherence was computed from the converted signals using MATLAB’s ‘mscohere’ function [38]. The time window of 4500 ns to 14,500 ns was chosen for the average MSC analysis, which represents the tail end of the signal. The limited length is based on studies related to coda wave interferometry, which typically uses a very short window at 3 ms or lower [26,28]. Typical coherence curves are shown in Figure 9. The ultrasonic signals from the different steel corrosion levels were each compared to the signals from the solid conditions (0% vs. 3%, 0% vs. 6%, 0% vs. 12% for D10 and D13 specimens, and 0% vs. 5%, 0% vs. 10%, 0% vs. 20% for D19 and D22 specimens). The coherence value was averaged within a certain frequency frame in this investigation so that the outcome may be reported as a single number and analyzed with steel corrosion levels. Two frequency ranges used for averaging MSC are shown in Figure 9. Ranges 1 and 2 represents frequencies lower than the central frequency of the transducers and frequencies adjacent (lower and higher) to the central frequency, respectively.

## 3. Recurrent Neural Networks (RNNs) of Ultrasonic Pulse Waves

### 3.1. Architecture

**Figure 10:**
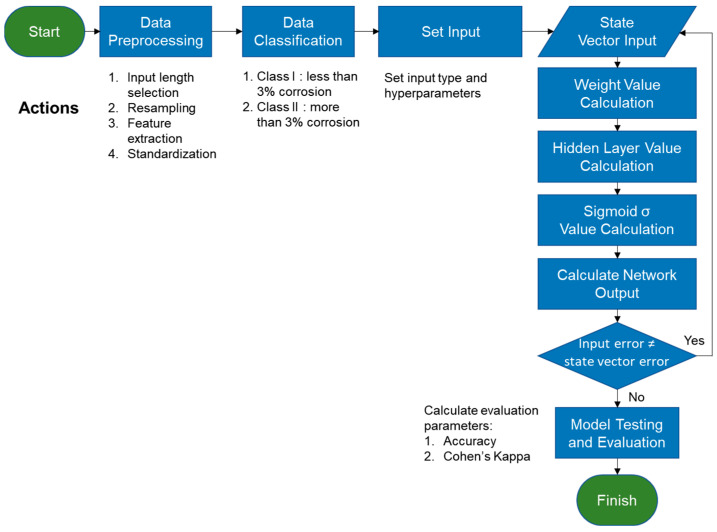
General flowchart of RNN model development.

**Figure 11:**
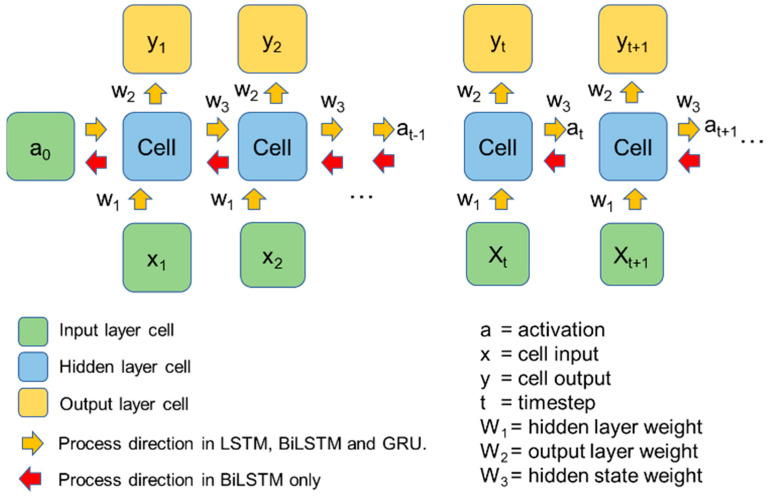
Arrangement of artificial neural network in deep learning.

The general flowchart of RNN development conducted in this study is shown in Figure 10. The RNN has the capability of analyzing time series data. This is because the RNN has the “memory” feature, called hidden state, that retains the information from the previous time steps. A typical RNN algorithm works as shown in Figure 11 in the following steps [39]: (1) the first data point in the sequence, *a_0_*, is delivered from the input layer to an RNN cell; (2) the cell adjusts the value of the a0 by a weighting function *w*, resulting as *y_1_*; (3) the second data in the sequence, *x_1_*, is put into the hidden layer; (4) both *x_1_* and *y_1_* are put together into the layer and have both of their output readjusted by weight; and (5) the process (2–4) is repeated until all of the data points within a sample have been processed in that layer and in the next hidden layers. The loss function, which is based on the errors obtained from every calculation in each layer, is then used to update the *w* from each hidden layer. The *w*, along with the algorithm, is updated from the last layer to the first. This process is called backpropagation [40] and is performed multiple times during the iteration until the least error value is obtained.

### 3.2. Long Short-Term Memory (LSTM)

**Figure 12:**
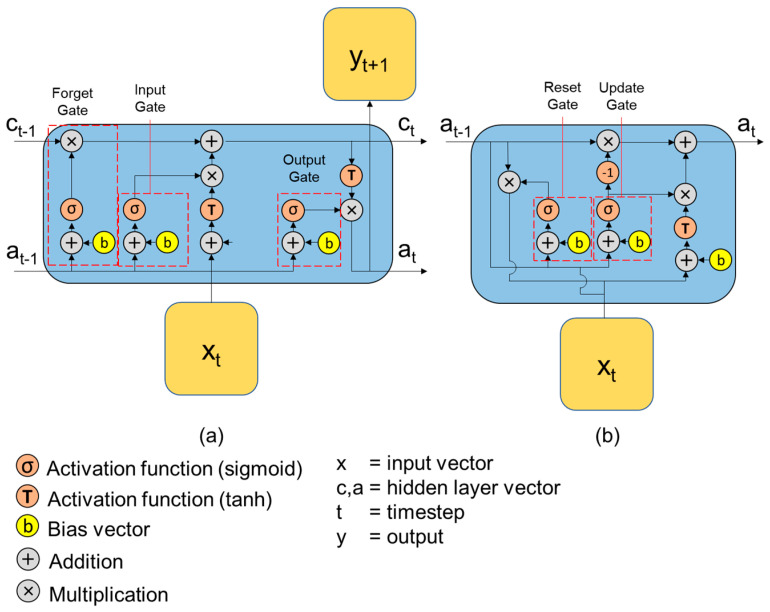
Architecture of a cell in the modified RNN methods for: (**a**) LSTM, and (**b**) GRU.

The primary form of a RNN might not give a satisfactory performance due to the vanishing gradient problem caused by long iterations [41]. Alternative approaches such as long short-term memory (LSTM) and gated recurrent unit (GRU) have been used to overcome the limitation of the RNN. The LSTM approach has internal gates to regulate information that should be retained or forgotten [42]. Figure 12a shows the architecture of an LSTM cell within an RNN and its working mechanism.

In general, LSTM works by the following steps: (1) selecting information to be discarded from the current input, (2) selecting information to be kept in the cell state, (3) updating the old cell information, and (4) determining state features of the output cell that should be retained. The work on the first step is conducted by the sigmoid unit that acts as the forget gate. In the second step, the *a*_*t*−*1*_ and *x_t_* are utilized to decide which information needs to be updated through the tanh layer, which effectively becomes the input gate. The *a*_*t*−*1*_ and *x_t_* are then used to go through the tanh layer to obtain the new cell information candidate *c_t_*. The third step updates the old information currently being kept in the cell or *c*_*t*−*1*_ based on the decision made by the forget gate (first step) and decided information candidate *c_t_* (second step). In the final step, the input *y*_*t*−*1*_ and *x_t_* are used by the sigmoid (output gate) to obtain a vector of values ranging from −1 to 1. These values are then multiplied by the weighting function from the output gate, resulting in the final output. The output of each gate in the LSTM is described in equations shown in Table 2, where *w_f_*, *w_i_*, *w_o_*, and *w_y_* are weighting functions and *b_f_*, *b_i_*, *b_o_*, and *b_h_* are bias vectors. It is also reported that having a bidirectional iteration of LSTM (or BiLSTM) can improve the model performance significantly [43]. This bidirectional approach basically uses the same principle as the conventional, forward-moving RNN, but with the process starting from the end part of the data in addition to the forward-moving RNN.

### 3.3. Gated Recurrent Unit (GRU)

Another widely used RNN method is the gated recurrent unit (GRU). Originally introduced by Cho et al. [44], GRU has recently been adopted by various researchers with a performance comparable to LSTM and, in some cases, outperforms LSTM [43,45]. For the same set of hyperparameters, the GRU network can also be trained faster since the architecture is simpler than LSTM: it has no cell state *c_t_* and instead uses the hidden state *y_t_* to transfer the information to the next cell. Furthermore, GRU only has two gates which are called the update gate and reset gate, as shown in Figure 12b. The equations in the GRU model are shown in Table 2, where *w* and *v* are the weight for *x_t_* and *a_t_*_−*1*_, respectively. With this simpler process, the GRU process takes less memory to compute and can be trained faster.

### 3.4. Data Preparation

The collected data was preprocessed to start with zero-mean, which improves learning efficiency of the RNN algorithms. The data was then prepared as two types: raw signal in the form of time series and extracted features in a systematic way. Both types of data were then downscaled into several sampling frequencies, which will be discussed further in Section 4.2, to further improve the training efficiency as well as to reduce the signal noises caused by a training process. The raw signal has a resolution equal to the sampling rate of the oscilloscope, which is 10 MHz. Considering that the central frequency of the transducers is 50 kHz, the maximum data resolution was limited to only 1 MHz to make the training process more efficient while still allowing higher frequency bands to be analyzed.

In this study, time series data were compressed by systematic feature extraction processes to further improve computational efficiency. For these purposes, standardized spectral entropy and instantaneous frequency were calculated to extract both time and frequency information from time series, following prior research works [46,47]. Spectral entropy is based on the equations of the power spectrum and probability distribution of signals. The power spectrum of the m^th^ signal (or *x*(*m*)) is denoted by *S*(*m*), where m is the index of frequency point. *S*(*m*) can be obtained by squaring the magnitude of its discrete Fourier transform *X*(*m*). Mathematically, the fundamental equations are as follows:

(7) $$H=-\overset{M}{\underset{m=1}{\sum }}R\left(m\right)log_{2}R\left(m\right)$$

(8) $$R\left(m\right)=\frac{S\left(m\right)}{\sum_{i=1}^{M}S\left(i\right)}$$

(9) $$S\left(m\right)={\left|X\left(m\right)\right|}^{2}$$

where *H* is the spectral entropy, *R*(*m*) is the probability distribution, *i* is frequency index in the calculation of *R*(*m*), and M is the total frequency points. The second feature is instantaneous frequency, which is a measure of change in the time parameter of a nonstationary signal associated with the average of frequencies as the signal changes. The fundamental equation of the instantaneous frequency $f_{inst}$ is as follows:

(10) $$f_{inst}\left(t\right)=\frac{\sum_{i=1}^{N}f_{i}P\left(t_{i},f_{i}\right)}{\sum_{i=1}^{N}P\left(t_{i},f_{i}\right)}$$

**Figure 13:**
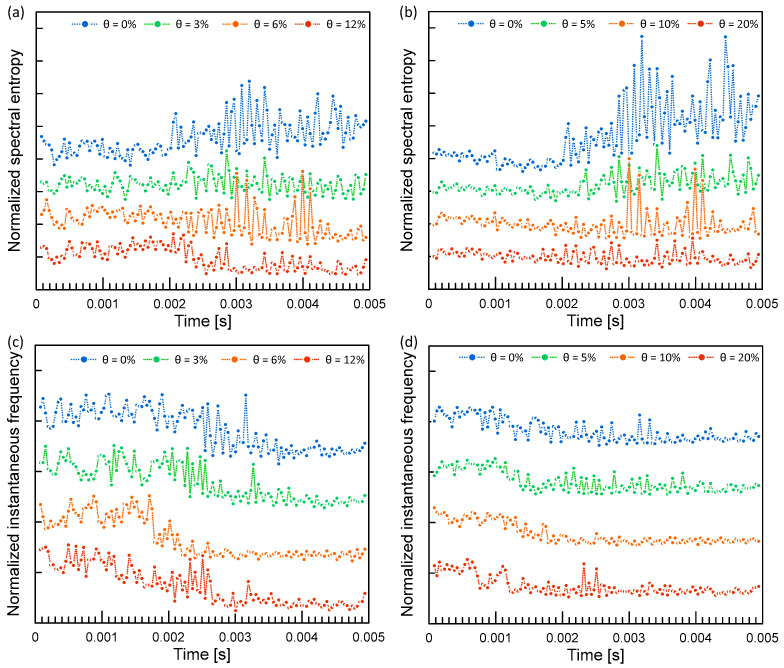
Typical feature extraction results for: (**a**,**b**) normalized spectral entropy, and (**c**,**d**) normalized instantaneous frequency of ultrasonic pulse waves measured from the concrete specimens with various corrosion levels. Left and right columns represent the results from the concrete specimens belonging to the MIX1 and D10, and the MIX1 and D19 groups, respectively.

Here, $f_{inst}$ is a summation of value from *i* = 1 to the N, where *i* is a step and N is the end step of the analyzed time-frequency window within a signal. Every step corresponds to the time step interval and its corresponding frequency measurement. P is the spectrogram power spectrum, *t* is time, and *f* is frequency of the signal input. More details regarding instantaneous frequency can be found in the research by Boashash [48]. Both spectral entropy and instantaneous frequency are standardized, which further improves the learning efficiency for neural network training [46]. The standardization is based on Z-scoring with the following equation applied to each data point in the dataset:

(11) $$Z=\frac{x-\mu }{\sigma }$$

where z is the new value on a data point after the standardization, *x* is the existing value of a data point, *μ* is the mean of dataset, and *σ* is the standard deviation. Figure 13 shows typical results from feature extraction. The feature extraction reduces the number of samples from 10,000 points in time series to 129 points, which significantly reduces computational cost.

### 3.5. Bilinear Classification Model

A bilinear classification model was developed to divide the dataset into two classes based on the steel corrosion level, *θ*, with a threshold of 3%. A part of the dataset corresponding to *θ* < 3% was classified as Class 1, which represents the solid to initial corrosion level. The remaining dataset (*θ* ≥ 3%) was classified as Class 2, which represents a medium to high corrosion level. A preliminary numerical experiment revealed that the effects of concrete mixture proportion and rebar size were not considerable on the performance of the classification model. In this study, ultrasonic pulse data were classified only based on steel corrosion levels (or concrete early damage condition). The more general model would be more effective as an in situ NDE method because specific material information is not always given or reliable in field surveys. The threshold level of the classification model was determined based on the initial steel corrosion level that starts to cause concrete deterioration (enhanced porosity and microcracking in concrete). In this study, it was observed that the relative mass of concrete specimens, which is the difference between damaged and solid concrete specimens, gradually increased as the amount of impressed current increased in the accelerated corrosion test. Based on that observation, it can be inferred that the expansion of rust products causes some internal concrete defects, which resulted in changes in the porosity of concrete. Surface-breaking cracks typically were observed at corrosion levels between 4% and 5%. Therefore, it is reasonable to say that a corrosion level of 3% can be selected as a threshold to detect early damage caused by steel corrosion in concrete. Based on the classification, there are a total of 222 data in Class 1 and 164 data in Class 2. RNN models were trained using 80% of data, randomly selected, from each class. The remaining 20% of the data for that class were used to test the trained model.

### 3.6. Performance Evaluation

The accuracy and Cohen’s kappa (later referred as kappa) were used to compare the classification performance of each method. The accuracy is a ratio between the true positive (*TP*) and true negative (*TN*) classifications given by the method to the total number of predictions [49]. Therefore, high numbers of false-positive (*FP*) and false-negative (*FN*) classifications will reduce network accuracy. However, accuracy alone is not enough to demonstrate a method’s performance. Researchers have incorporated kappa to evaluate prediction models [50,51], in addition to the accuracy. The kappa is a chance-corrected method for assessing agreement among raters [52]. The equations to calculate these parameters for this study are as follows:

(12) $$Accuracy=\frac{TP+TN}{TP+TN+FP+FN},\;\mathrm{and}$$

(13) $$Cohen’s\;Kappa=\frac{\mathrm{Accuracy}+p_{e}}{1-p_{e}}.$$

where $p_{e}$ is the rate of agreement between the prediction and actual class value by chance, which is calculated using following equation,

(14) $$p_{e}=\frac{\left(\left(TN+FN\right)\times \left(TN+FP\right)\right)+\left(\left(FP+TP\right)\times \left(FN+TP\right)\right)}{\left(TP+TN+FP+FN\right)}.$$

with Kappa ranges from −1 to 1, with the value below 0.4 regarded as low agreement, 0.41 to 0.60 regarded as moderate agreement, 0.61 to 0.80 as substantial agreement, and 0.81 to 0.99 as near-perfect agreement [53].

## 4. Results and Discussion

### 4.1. Bilinear Classification Models Based on Conventional Ultrasonic Testing Parameters

Bilinear classification models were developed based on the two conventional ultrasonic testing parameters, which will be compared with deep learning classification models in Section 4.3. In this study, two conventional ultrasonic testing parameters (relative P-wave velocity and signal consistency) were reduced from the ultrasonic pulse waves collected from the concrete specimens with different steel corrosion levels, *θ*. The two reduced ultrasonic testing parameters were divided into two classes according to the steel corrosion level, with a threshold of 3%, consistent with the definition described in Section 3.5 for the deep learning classification models.

**Figure 14:**
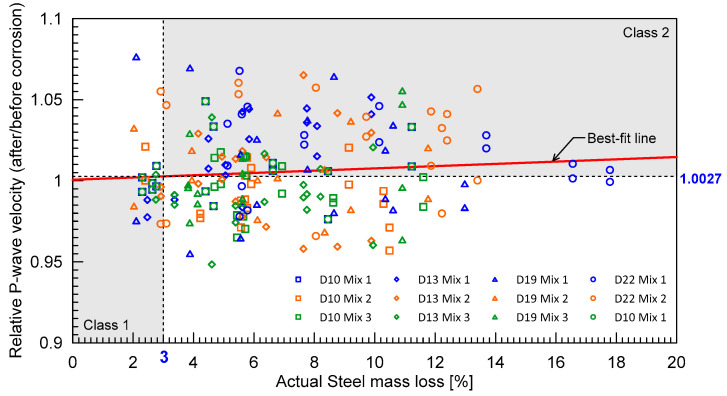
Classification based on the relative P-wave velocity data.

Figure 14 shows the distribution of the relative P-wave velocity (i.e., the P-wave velocity of damaged concrete after the accelerated corrosion process normalized by the P-wave velocity of solid concrete) with actual steel corrosion levels. Overall, the effect of the steel corrosion levels cannot be clearly seen because of a scattering of the relative P-wave velocity with steel corrosion levels from 0% to 20%. In this study, a linear equation was used to relate the relative P-wave velocity and the steel corrosion levels as follows:

(15) $$V_{r,P}=0.0007\theta +1.0006,\;with\;R^{2}=0.0008$$

where *V_r_*_,*P*_ is the relative P-wave velocity of concrete, and θ is the actual (measured) steel mass loss (or corrosion level). The best-fit line looks almost flat with particularly low *R*^2^, which reveals that the relative P-wave velocity might not be an effective parameter for evaluating concrete damages caused by corrosion of steel in concrete.

**Figure 15:**
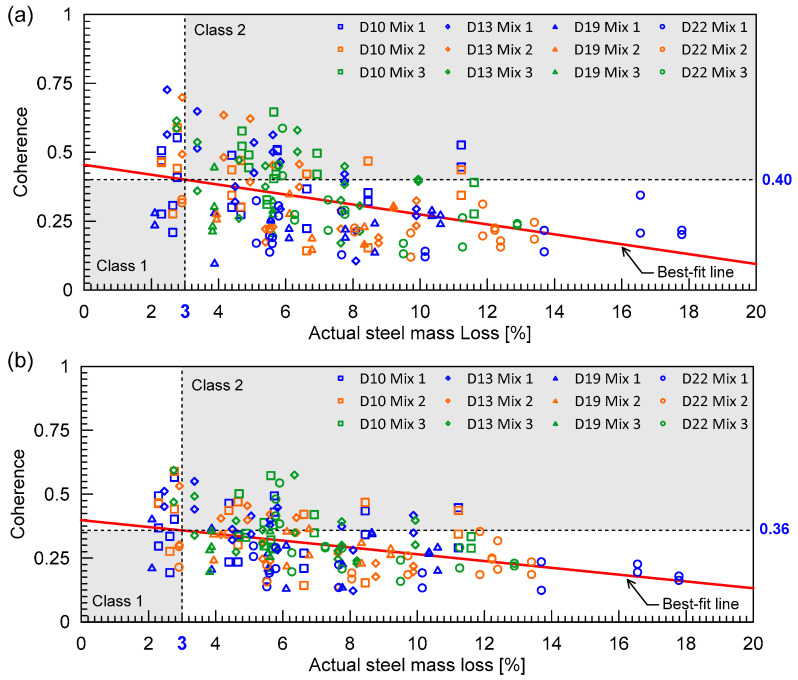
Classification based on the average MSC method using two different frequency ranges: (**a**) Range 1, and (**b**) Range 2 (see Figure 9).

Figure 15a,b show the distribution of average magnitude square coherence (MSC) values for the two different frequency ranges: Range 1 and 2, respectively. Average MSC shows a descending trend with respect to the steel mass loss. It can be seen that average MSC is a better indicator for classifying early concrete damage caused by steel corrosion than *V_r_*_,*P*_. Using linear regression, the obtained *R*^2^ were 0.18 and 0.17 for Range 1 and Range 2, respectively. The regression equations for Range 1 and Range 2 were obtained as follows:

(16) MSC1=−0.0166θ+0.4523 for Range 1

(17) MSC2=−0.0133θ+0.3983 for Range 2

Table 3 summarizes the threshold values for *V_r_*_,*P*_, and average MSCs was determined by the linear regression equations (Equations (15)–(17)), corresponding to the steel corrosion level of 3%. The confusion matrix based on the classification models for the two conventional ultrasonic testing parameters is summarized in Table 4. Accuracy and Cohen’s kappa values were calculated using Equations (12)–(14). As expected, the performance of the classification model based on relative P-wave velocity, *V_r_*_,*p*_, was not satisfactory, with accuracy of 53% and kappa of 0.07. Those values are as low as those predicted by chance alone. Conversely, the classification models based on MSC resulted in far more improved accuracies greater than 70%. The improved accuracy demonstrates that ultrasonic pulse waves can be used for early detection of concrete damages caused by steel corrosion. It can be inferred that incoherence parts of ultrasonic pulse waves are more sensitive to the internal concrete damages by progression of steel corrosion than coherence parts. However, it should be noted that the performance of the MSC-based models, like other nonlinear ultrasonic parameters, is strongly affected by engineering + judgment on the selection of input signals and signal processing processes. For example, two different frequency ranges (Ranges 1 and 2) in this study resulted in different performances (Table 3). Therefore, more systematic approaches are needed to optimize the capabilities of ultrasonic pulse wave measurements for condition assessment of concrete.

### 4.2. Deep Learning Classification Model

A series of numerical experiments was performed to investigate the variation of the performance of deep learning classification models, with varying inputs and hyperparameters for RNN models. As such, an optimal set of parameters was determined, which resulted in the best performance of the deep learning classification model. Table 5 summarizes the critical parameters and ranges of each parameter considered in this study.

#### 4.2.1. Effects of Input Type

**Figure 16:**
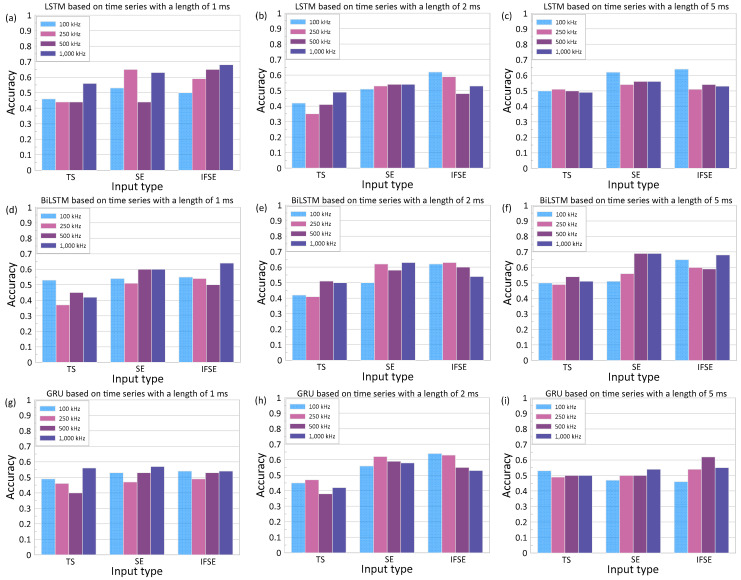
Variation of accuracy of the deep learning classification models based on three input data types (time series, TS; spectral entropy, SE; and combination of instantaneous frequency and spectral entropy, IFSE). Top, middle, and bottom rows represent the results based on the three RNN models: LSTM, BiLSTM, and GRU, respectively. Left, middle, and right columns represent the results based on three different lengths of ultrasonic pulse waves: 1 ms, 2 ms, and 5 ms, respectively.

To observe the effect of input type on the model performance, a series of trainings using different types of input has been conducted. The input consists of (1) time series (TS), which is the raw signal in the time domain, (2) spectral entropy (SE), and (3) a combination of instantaneous frequency and spectral entropy (IFSE). Figure 16 shows the variations of accuracy of the deep learning classification models with these inputs based on the three RNN models (i.e., LSTM, BiLSTM, and GRU). While not shown in this article, it is found that the use of IF as an input in the RNN models resulted in similar trends to those based on SE. The top, middle, and bottom rows of the figure are results for LSTM, BiLSTM, and GRU, respectively. The left, middle, and right columns of the figure are the results using time series data with lengths of 1 ms, 2 ms, and 5 ms, respectively. Training of the RNN models was performed with fixed hyperparameters, epoch of 100 and batch size of 16. Hyperparameters will be discussed in more detail in Section 4.2.3. In addition, the Adam optimizer and a network size of 100 were used as default values in the deep learning toolbox in MATLAB.

Overall, the use of SE and IFSE resulted in comparable accuracy levels. It was also observed that the use of IFSE for fixed networks and length of time resulted in slightly better accuracy values compared to the models based on SE. The best accuracy was 69%, which was obtained based on SE of ultrasonic pulse waves with a length of 5 ms and a sampling frequency of 1000 kHz, as a base input of the deep learning model. The accuracy based on IFSE was 68% for the same test setup (see Figure 16f). It can be noticed that the accuracy of the deep learning classification models based on an input of time series (TS) was less than that of using spectral entropy (SE) or the combination of spectral entropy and instantaneous frequency (IFSE). The best accuracy based on TS was only 56% for the LSTM model using an input of TS with a length of 1 ms and a sampling frequency of 1000 kHz. For the same set of parameters, the use of SE and IFSE resulted in more improved accuracy of 63% and 68%, respectively.

Care is needed when using the time series data as an input of RNN models of ultrasonic pulse waves in concrete. Ultrasonic pulse wave data collected in this study involved relatively high variabilities in the amplitude and first arrival times. The variability of time series data could be caused by various sources of experimental uncertainties, such as inconsistent coupling conditions, surface roughness of concrete and heterogeneous features of concrete, which are not really related to the severities of concrete damages. RNN models are known to be especially sensitive to the sequence of the data points. Consequently, experimental uncertainties in ultrasonic waves could have more impact on the performance of the RNN-based classification models [54]. Moreover, RNN models trained using the time series data have high computational costs. Therefore, it is not recommended to use time series data as an input for RNN-based deep learning in this study.

#### 4.2.2. Effects of Input Data

**Figure 17:**
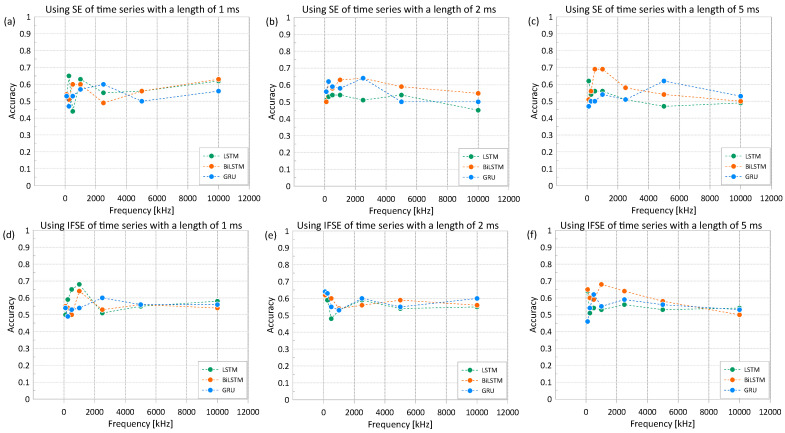
Variation of accuracy of the deep learning classification models based with various sampling frequencies for calculation of inputs. Top and bottom rows represent the results based on the SE and IFSE, respectively. Left, middle, and right columns represent the results based on the three different lengths of ultrasonic pulse waves: 1 ms, 2 ms, and 5 ms, respectively.

**Figure 18:**
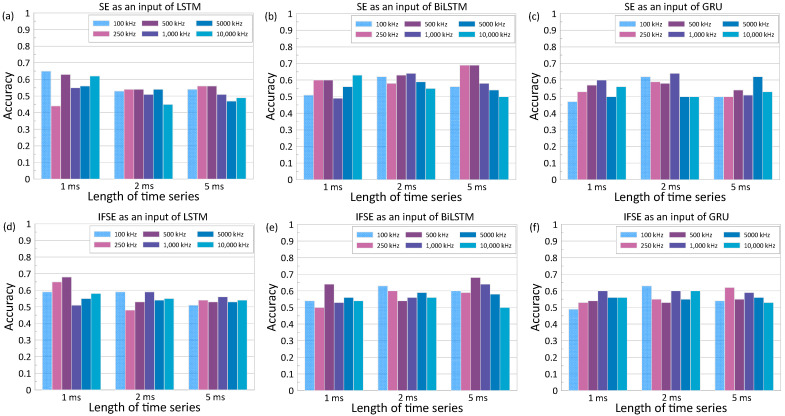
Variation of accuracy of the deep learning classification models based on three different lengths of time series for calculation of inputs. Top and bottom rows represent the results based on the SE and IFSE, respectively. Left, middle, and right columns represent the results based on the three different RNN networks: LSTM, BiLSTM, and GRU, respectively.

Figure 17 and Figure 18 show the variations of the accuracy of the deep learning classification models with increasing sampling frequency of ultrasonic pulse waves used for the calculation of input data for RNN models. These figures represent the results based on SE and IFSE extracted from ultrasonic pulse wave data with different lengths (1 ms, 2 ms, and 5 ms). Training of the models was performed with fixed hyperparameters, epoch of 100 and batch size of 16. The Adam optimizer and network size of 100 were used as default values in toolbox in MATLAB. Overall, it was noticed that the best accuracy of each model was obtained at a sampling frequency lower than 1.0 MHz. This is reasonable since the use of 50 kHz transducers in this study resulted in an effective bandwidth with a frequency range of 100 kHz to 1000 kHz. The best accuracy was observed at 69% by using SE of ultrasonic pulse waves with a length of 5 ms and a sampling frequency of 500 kHz as an input of BiLSTM (see Figure 17c).

In addition to sampling frequency, the variation of signal length was also considered. The networks were trained with 5 ms (full length), 2 ms, and 1 ms. It is noticed that each network behaves differently to the input data properties. The LSTM network performed better using shorter sample lengths, with the best accuracy of 68%. The accuracy of LSTM models increases from 54% to 65% as the length of signals decreases from 5 ms to 2 ms. It can be inferred that the LSTM is more sensitive to the coherent part of the signal, which is mostly located in the early part of the time series. On the other hand, the accuracy of the model based on GRU remained stagnant without regard for the length of time series, with the best accuracy in a range of 60% to 64%. The most noticeable improvement was found with the use of BiLSTM: the longer time series resulted in greater accuracy. The accuracy of the BiLSTM classification models increased from 62% to 69% as the length of times signals used for calculation of IFSE increased from 1 ms to 5 ms.

#### 4.2.3. Effects of Hyperparameter

This section discussed the effect of the hyperparameter setup, particularly related to the number of epochs and batch sizes, on the accuracy of the deep learning models. Deep learning models, including RNNs, can be run with virtually limitless combinations of hyperparameters. Models that are undertrained often have the characteristics of unbalanced true predictions between each class, which translates to low accuracy. On the other hand, models that are overtrained often cannot recognize the dataset outside of the training dataset, leading to overfitting. In an RNN, an epoch refers to a single pass through the entire dataset during training. During each epoch, the model’s parameters are updated based on the errors made in predicting the output for each example in the dataset. The batch size refers to the number of sequences that are processed simultaneously by the network during training. The batch size is adjusted to balance the trade-off between computational efficiency (training time) and the ability to estimate the true gradient of the loss function.

**Figure 19:**
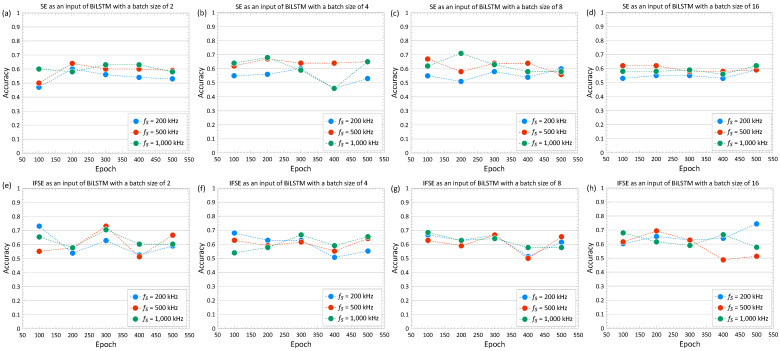
Variations of accuracy with epochs in a range of 100 to 500 with various batch sizes and two different types of input for BiLSTM. The upper and lower rows indicate the results based on spectral entropy (SE) and combination of instantaneous frequency and spectral entropy (IFSE), respectively. The first to fourth columns from the left side indicate the results determined by using batch sizes of 2, 4, 8, and 16, respectively. The results from three different sampling frequencies (250 kHz, 500 kHz, and 1000 kHz) are presented as red, blue, and green solid circles in each figure.

Figure 19 shows the variation of accuracy of the deep learning classification model with five different epochs (100, 200, 300, 400, and 500) and four different batch sizes (2, 4, 8, and 16). All results in the figure were calculated from BiLSTM based on SE or IFSE as an input. SE and IFSE were calculated based on the time series with a length of 5 ms. The results from three different sampling frequencies (250 kHz, 500 kHz, and 1000 kHz) are shown in each figure. Overall, it was observed that the IFSE input resulted in slightly higher accuracy than the models trained only with SE. For most results from the use of SE and IFSE, accuracy of the RNN model changes only little with the various epochs and batch sizes considered in this study. However, it was clearly noticeable that the accuracy of the classification model could be enhanced by tunning the hyperparameters. For example, the use of SE results in best accuracy of 71% for the parameter setup of sampling frequency of 1000 kHz with epoch of 200 and batch size of 8. Furthermore, the use of IFSE results in slightly higher best accuracy of 74% for the parameter setup of sampling frequency of 250 kHz with epochs of 500 and batch size of 16. The accuracy of 74% on that setup was the best-attained accuracy in our tests. This reveals that the deep learning classification model based on BiLSTM can improve the accuracy of the classification up to 150% more than the estimation by chance alone.

### 4.3. Performance Comparison of Methods

**Figure 20:**
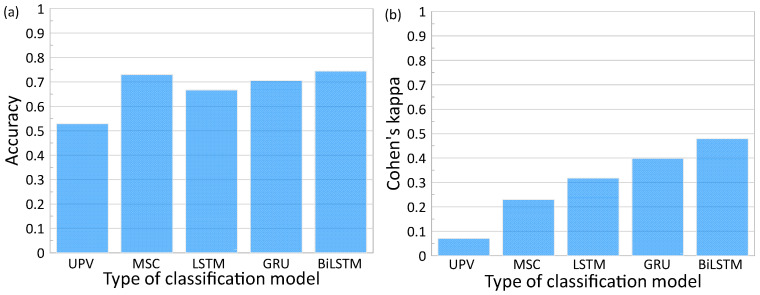
Comparison of performance of classification models based on all tested methods: (**a**) accuracy, and (**b**) Cohen’s kappa.

Figure 20 compares the best performance of the deep learning classification models based on the three RNN models (LSTM, GRU, and BiLSTM) with tuned hyperparameters. Furthermore, the performance of the classification models based on the two conventional ultrasonic parameters (relative P-wave velocity and signal coherence) is also shown in the figure. The classification thresholds for the conventional methods are based on the obtained regression lines with θ at 3%, which returns $V_{r,P\;3\%}$ of 1.00027 and coherence of 0.36.

It was demonstrated that the performance of the deep learning classification models was far more improved than those based on the relative P-wave velocity, *V_r_*_,*p*_. Among the tested parameters, the BiLSTM model with fine-tuned hyperparameter has the best overall performance, with an accuracy of 74% and kappa of 0.48. The GRU model has slightly less performance, with the top performing model demonstrating an accuracy of 71% and kappa of 0.40. The least performing RNN model was the LSTM with an accuracy of 67% and kappa of 0.32. The UPV method was placed in a distant last place, with an accuracy of 53% and kappa of 0.07. Therefore, *V_r_*_,*p*_ is not an effective parameter for detecting early concrete damages caused by steel corrosion in the rust propagation period. Previous researchers observed that UPV of concrete was sensitive to the presence of surface-breaking cracks and subsurface cracks and voids [55]. However, *V_r_*_,*p*_ of concrete did not show a clear correlation with the corrosion levels of steel in concrete in this study, even for the concrete specimens with surface-breaking cracks (i.e., D19 and D22 specimens). It was observed in this study that the surface-breaking cracks first appeared on the surface of concrete at a corrosion level ranging from 4% to 5%. The surface-breaking cracks were tightly closed at the early stage of corrosion. Furthermore, corrosion products could fill the microcracks and enhanced porosity of damaged concrete. Therefore, it can be inferred that early concrete damages caused by steel corrosion could not affect the coherence parts of ultrasonic pulse waves. Even so, the signal-coherence-based classification model resulted in far more improved accuracy of 73% compared to the UPV model. This result reveals that incoherent parts of ultrasonic pulse waves are informative of the minor concrete damages associated with steel corrosion. However, the balance of true positive predictions between the classes from the signal coherence method was relatively low, which resulted in a kappa of 0.23. The overall low performance of the signal coherence method is attributed to the significant changes in frequency response caused by the formation of cracks in concrete. The variation of signal coherence remains a very low value and becomes stable regardless of the corrosion level progress. Furthermore, as discussed in this study, the distribution of signal coherence, as a nonlinear ultrasonic parameter, could be dependent on several signal processing parameters such as the length of signals, the range of time, and frequency windows, as discussed in Section 4.1. Therefore, special cares are needed to find an optimal set of parameters that result in the best performance of the model based on signal coherence. Therefore, it was demonstrated that the deep learning approach based on RNN for ultrasonic pulse waves is a potential method for evaluating early concrete damage caused by steel corrosion in the rust propagation stage.

## 5. Conclusions

This study investigated the feasibility of ultrasonic pulse wave measurements for early detection of concrete damages caused by corroded steel in concrete using a deep learning approach based on RNN. A series of experimental studies was performed in the laboratory to collect ultrasonic pulse waves through reinforced concrete cube specimens where a reinforcing steel with various corrosion levels was embedded. A bilinear model, classifying the reinforced concrete cubes into solid and damaged concrete according to the threshold of 3% steel corrosion, was developed based on deep learning of ultrasonic pulse waves using RNN. The performance of the deep learning classification model based on three different RNN models (LSTM, GRU, and BiLSTM) were compared. Furthermore, the performance of the deep learning classification models were compared with the classification models based on two conventional ultrasonic testing parameters (relative P-wave velocity and signal consistency). Summarized below are four important findings in this study:

1. The performance of deep learning classification models was affected by various parameters: length of time signal, sampling frequency of time signal, type of input, networks, and hyperparameters (batch size and epoch). The use of an extracted feature (i.e., IF and/or SE) as an input of RNN-based deep learning models resulted in better performance and far more improved computational efficiency than using time series. It was observed that time series with a length of 5 ms and a sampling frequency of 500 MHz was appropriate as an input of the feature extraction processes. However, it was difficult to reach general conclusions on the effects of various input and training parameters because different sets of parameters affected the performance results for the three RNN algorithms in a different way. A fine-tuning of hyperparameters further improved the performance of deep learning classification models.
2. Deep learning classification models, in the form of RNNs, were effective for the evaluation of early concrete damage caused by steel corrosion in concrete with acceptable accuracy. The best performance was obtained using the bidirectional long short-term memory (BiLSTM) model based on a combination of instantaneous frequency and spectral entropy (IFSE) of ultrasonic pulse waves with a length of 5 ms and a sampling frequency of 250 kHz. Based on this method, the obtained accuracy and Cohen’s kappa were 74% and 0.48, respectively.
3. It was demonstrated that the relative P-wave velocity was not practical for detecting early concrete damages associated with steel corrosion in the rust propagation phase. The classification model based on ultrasonic pulse velocity (UPV) resulted in a relatively low accuracy of 53% with a kappa of 0.07.
4. The classification model based on signal coherence achieved more improved accuracy of 73% than that based on UPV. However, the method resulted in a relatively low kappa of 0.23, which is attributed to unbalanced true positives between classes. In addition, there are many parameters in this method, such as the analyzed signal time series and frequency window, that are not standardized and require engineering judgment. Therefore, special care is needed to optimize the model that results in the best performance of the model.
5. The ultrasonic pulse wave data used in this study were collected from the limited number of concrete specimens fabricated in the laboratory, with corroded steel artificially accelerated by the impressed current technique, in specific saturation conditions (fully saturated conditions). Furthermore, signal measurements in this research were performed in the direct measurement configuration, which could limit this study’s practicality for actual structures. Therefore, more systematic studies that consider various experimental conditions in field applications are needed to reach more general conclusions.

## Figures and Tables

**Table 1:** Summary of mixture proportions of concrete used for fabrication of concrete cube specimens in this study.

Mix Design	w/c (%)	Mixture Proportion (kg/m^3^)				
		W	C	S	G	AE
Mix 1	58.5	168	287	957	898	2.58
Mix 2	50.7	170	335	870	956	2.50
Mix 3	34.6	166	480	720	993	4.32

Note: W: water, C: cement, S_V_: volume of sand, A_V_: volume of aggregates, C: Portland cement type I, S: sand, G: gravel, AE: high-performance air-entraining agent.

**Table 2:** Outputs in an LSTM and a GRU cell.

Model	Gate Type	Equation
LSTM	Forget gate	$f_{t}=\sigma \left(w_{f}\left[a_{t-1},x_{t}\right]+b_{f}\right)$
	Input gate	$i_{t}=\sigma \left(w_{i}\left[a_{t-1},x_{t}\right]+b_{i}\right)$
	Output gate	$o_{t}=\sigma \left(w_{o}\left[a_{t-1},x_{t}\right]+b_{o}\right)$
	Hidden gate	$y_{t}=tanh\left(w_{c}\left[a_{t-1},x_{t}\right]+b_{y}\right)$
	Final Output	$c_{t}=f_{t}c_{t-1}+i_{t}y_{t}$ $a_{t}=o_{t}\;tanh\;c_{t}$
GRU	Update gate	$z_{t}=\sigma \left(w_{z}x_{t}+v_{z}a_{t-1}+b_{z}\right)$
	Reset gate	$r_{t}=\sigma \left(w_{r}x_{t}+v_{r}a_{t-1}+b_{r}\right)$
	Hidden state	$y_{t}=tanh\left(w_{c}\left[a_{t-1},x_{t}\right]+b_{y}\right)$
	Final output	$a_{t}=z_{t}.a_{t-1}+\left(1-z_{t}\right)y_{t}$

**Table 3:** Parameter thresholds for *V_r_*_,*p*_ and MSC methods.

Class	Steel Corrosion Loss	*V_r_*_,*p*_	*MSC1*	*MSC2*
Class 1 (N)	<3%	*V_r_*_,*p*_ < 1.0027	MSC > 0.40	MSC > 0.36
Class 2 (P)	≥3%	*V_r_*_,*p*_ ≥ 1.0027	MSC ≤ 0.40	MSC ≤ 0.36

**Table 4:** Confusion matrix for classification based on *V*_*r*,*p*_ and MSC methods.

		Predicted					
		*V_r_*_,*p*_		*MSC1*		*MSC2*	
		P	N	P	N	P	N
**Actual**	**P**	95 (45.2%)	91 (43.4%)	119 (62.9%)	45 (23.8%)	123 (65.1%)	41 (21.7%)
	**N**	8 (3.8%)	16 (7.6%)	10 (5.3%)	15 (7.9%)	10 (5.3%)	15 (7.9%)
Accuracy		52.8%		71.0%		73.0%	
Cohen’s kappa		7%		20.0%		22.9%	

**Table 5:** Summary of critical parameters as variables for training deep learning models of ultrasonic pulse waves in this study.

Parameter		Range
Length of time signal		1 ms, 2 ms, and 5 ms
Sampling frequency of time signal		100 kHz, 250 kHz, 500 kHz, and 1000 kHz
Type of input		Time series (TS), instantaneous frequency (IF), spectral entropy (SE), and combination of IF and SE (IFSE)
Network		LSTM, BiLSTM, and GRU
Hyperparameter	Batch size	1, 2, 4, 8, and 16
	Epoch	100, 200, 300, 400, and 500

## Data Availability

Not applicable.
